# Presence of autoantibodies targeting the shared epitope in rheumatoid arthritis and psoriatic arthritis

**DOI:** 10.3389/fimmu.2026.1744370

**Published:** 2026-03-03

**Authors:** Eduard Graell, Juan Francisco Delgado, Antonio Domingo Gomez, Maria García-Marique, Menna Rusiñol, Albert Rodrigo, Antoni Berenguer-Llergo, Jordi Gratacos, Joan Calvet

**Affiliations:** 1 Rheumatology Department, Parc Taulí Hospital Universitari, Sabadell, Spain; 2 Institut d’Investigació i Innovació Parc Taulí (I3PT-Centres de Recerca de Catalunya (CERCA)), Universitat Autònoma de Barcelona, Sabadell, Spain; 3 Inflammatory Joint Diseases, Bone Metabolism, and Systemic Autoimmune Diseases Research Group, Institut d’Investigació i Innovació Parc Taulí (I3PT-CERCA), Sabadell, Spain; 4 Immunology Laboratory, Clinical Laboratories Service, Parc Taulí Hospital Universitari, Sabadell, Spain

**Keywords:** anti-citrullinated peptide antibodies, autoantibodies, inflammatory arthritis, rheumatoid arthritis, shared epitope

## Abstract

**Introduction:**

Rheumatoid arthritis (RA) is an autoimmune disease marked by the production of autoantibodies (AAb) against citrullinated proteins/peptides (ACPA). The shared epitope (SE) in the HLA-DRB1 gene is a major genetic risk factor for RA and has been linked to ACPA production, particularly in individuals exposed to environmental triggers such as smoking. However, the role of the SE itself, especially in its citrullinated form, as a target of humoral immunity in RA remains underexplored.

**Methods:**

We analyzed autoantibodies against SE-containing peptides (SE-AAb) in 150 RA patients, 62 patients with psoriatic arthritis (PsA), and 204 healthy controls. HLA-DRB1 polymorphisms associated with the SE (QKRAA, QRRAA, RRRAA) were evaluated by PCR-SSO. IgG reactivity against native, citrullinated, and carbamylated SE peptides, in linear and cyclic conformations, was assessed using a custom ELISA.

**Results:**

SE-AAb were detected in RA patients with frequencies ranging from 26.0% to 45.3%, depending on peptide conformation and post-translational modification, with the highest positivity observed against citrullinated SE peptides. Antibodies against cyclated citrullinated SE peptides were present in 45.3% of RA patients, compared with 21.6% of healthy controls. PsA patients also showed SE-AAb positivity, with frequencies ranging from 17.7% to 35.5%, displaying a pattern largely similar to RA. No significant association was observed between SE-AAb positivity and SE genetic carriage, and SE-AAb presence was not associated with clinical features of RA.

**Conclusion:**

SE-AAb are present in RA patients, particularly against citrullinated SE peptides, but are not specific to RA, as similar reactivity is observed in PsA. The presence of these autoantibodies is independent of SE genetic carriage, suggesting that inflammatory conditions rather than genetic SE status may contribute to their generation. Further studies are needed to clarify the clinical relevance of SE-AAb in RA pathogenesis.

## 1 Introduction

Rheumatoid arthritis (RA) is an autoimmune disease characterized by excessive production of autoantibodies (AAb). This results from dysregulation of the immune response in genetically predisposed individuals exposed to environmental factors such as smoking or periodontitis (1–4).

Among the AAb described in RA, anti-citrullinated protein/peptide antibodies (ACPA) are the most relevant. They are highly specific for RA, form part of the disease classification criteria, and are associated with a more aggressive clinical course (1, 5–7). Studies in population biobanks have shown that ACPAs can be detected decades before the clinical onset of RA (8–10). Initially, IgM is the predominant ACPA isotype. The subsequent appearance of IgA and IgG correlates with clinical onset and established disease, respectively (9, 10). These observations underscore the central role of ACPAs in RA pathophysiology (10, 11).

ACPA production is influenced by genetic factors associated with RA, most notably the shared epitope (SE), the main genetic risk factor for the disease. SE carriage has been strongly associated with ACPA positivity, particularly in individuals exposed to smoking (12, 13). In addition, periodontitis has been linked to ACPA presence, further supporting the interaction between genetic susceptibility and environmental exposures in RA (14).

ACPAs were initially described as targeting citrullinated filaggrin, an extra-articular protein (15). Subsequently, several intra-articular citrullinated proteins were identified, including fibrin and fibrinogen (16–18). These proteins share peptide sequences with filaggrin (19). Additional autoantibodies against citrullinated peptides and proteins have been described, such as those targeting fibronectin, alpha-enolase, and vimentin. However, their clinical utility is limited due to low sensitivity (9, 18, 20–32).

Currently, the most widely used commercial assay for RA detection is based on cyclic citrullinated peptides (CCP3). This test shows a specificity of 86–99% and a sensitivity of 60–78% (17, 33). However, because CCP3 relies on synthetic peptides, a single human citrullinated protein determinant directly involved in RA pathophysiology has not yet been identified.

The SE is a polymorphism in the HLA-DRB1 gene and represents the genetic factor with the greatest impact on RA susceptibility (34). It encodes a conserved five–amino acid sequence (QKRAA, QRRAA, or RRRAA) located in pocket 4 of the HLA class II molecule. Several studies have shown that this motif confers increased affinity for the presentation of citrullinated peptides (32, 35–37).

The SE sequence contains arginine residues that can be citrullinated by peptidyl arginine deiminase (PAD) enzymes (38). We hypothesized that the SE, particularly in its citrullinated form, may function as an antigen capable of inducing ACPA production in RA. During inflammation, cell death and protein degradation occur alongside PAD activation. Under these conditions, the SE peptide may undergo citrullination. Exposure of this peptide to PAD during inflammatory proteolysis could generate citrullinated sequences capable of eliciting autoantibody responses. Alternatively, citrullination within the HLA class II structure itself could alter molecular conformation and modulate immune recognition.

Despite the strong genetic association between the shared epitope and ACPA production, it remains unclear whether the shared epitope itself represents a direct target of humoral autoimmunity in RA, particularly in its native or post-translationally modified forms. In this study, we therefore investigated the presence of autoantibodies directed against different shared epitope sequences (SE-AAb), in both native and post-translationally modified forms, in patients with RA.

## 2 Materials and methods

### 2.1 Study design and participants

This single-center, cross-sectional study analyzed the presence of SE-AAb in a cohort of patients with RA and evaluated its association with the SE and baseline clinical characteristics. Additionally, the prevalence of SE-AAb in the RA cohort was compared with that observed in two control cohorts: one comprising patients with psoriatic arthritis (PsA), and the other consisting of healthy blood donors from the Banc de Sang i Teixits of Barcelona. Donor samples were provided in an anonymized format, and individual-level demographic variables, including age and sex, were not available to the investigators for the control cohort. Given the limited prior data on shared-epitope autoantibodies, this study was designed as an exploratory analysis and no formal sample size calculation was performed.

All RA patients included in the study were recruited from the outpatient clinics of Parc Taulí University Hospital (PTUH). Eligible patients met the 2010 EULAR/ACR classification criteria for rheumatoid arthritis and were over 18 years of age. A systematic retrospective review of medical records was carried out between June and October 2022. When available, data were collected on smoking history, previous and current treatments, radiographic evidence of joint involvement in the hands and feet, and serological markers, including rheumatoid factor (RF), ACPA, and antinuclear antibodies.

All participants, including RA patients, PsA patients, and healthy blood donors, had signed informed consent to participate.

The present study was reviewed and approved by the Ethics and Drug Research Committee of PTUH (Ref.: 2018/534) in accordance with the World Medical Association Declaration of Helsinki.

### 2.2 HLA typing

HLA-DRB1 polymorphisms were evaluated in RA patients and PsA patients. HLA-DRB1 gene polymorphisms were analyzed using PCR-SSO with the LIFECODES HLA-DRB1 eRES SSO Typing Kit (Immucor, Norcross, GA, USA). We defined the shared epitope alleles in our study as the HLA-DRB1*01:01, HLA-DRB1*01:02, HLA-DRB1*04:01, HLA-DRB1*04:04, HLA-DRB1*04:05, HLA-DRB1*04:08, HLA-DRB1*10:01, HLA-DRB1*14:02 (39).

### 2.3 Rheumatoid arthritis serological markers

ACPAs were measured using the QUANTA Flash^®^ CCP3 chemiluminescence immunoassay (Inova Diagnostics, San Diego, CA, USA), and RF was assessed using a turbidimetric immunoassay (The Binding Site, Birmingham, UK).

### 2.4 Autoantibodies against the shared epitope

Serum samples from 150 RA patients, 62 PsA patients, and 204 healthy controls were tested using a custom ELISA with a 13-amino acid peptide sequence from positions 66–78 of HLA-DRB1, containing the three SE sequence variants (native SE, citrullinated SE, and carbamylated SE), synthesized in both linear and cyclic forms, except for the carbamylated peptide, synthesized in its linear form.

Immulon 4 HBX plates (Nunc, Thermo Fisher Scientific, Waltham, MA, USA) were coated with 10 μg/mL of recombinant SE peptides (ProImmune, Oxford, UK) in a 50 mM carbonate-bicarbonate buffer (pH 9.6) and incubated overnight at 4 °C. Subsequently, the plates were blocked with 5% PBS-BSA (Sigma-Aldrich, St. Louis, MO, USA) for 2 hours at room temperature. Serum samples were then diluted 1:100 in PBS-Tween 20^®^ (Sigma-Aldrich, St. Louis, MO, USA) and incubated for 1 hour at room temperature. After washing, HRP-conjugated anti-human IgG (Thermo Fisher Scientific, Waltham, MA, USA) diluted 1:2,000 in PBS-Tween 20^®^ was added and incubated for 1 hour at room temperature. The enzymatic reaction was developed using tetramethylbenzidine (Thermo Fisher Scientific, Waltham, MA, USA) for 30 minutes at room temperature and stopped with 25% sulfuric acid (Merck, Darmstadt, Germany). Absorbance was measured at 450 nm, using 620 nm as the reference filter. The cutoff value was established to ensure 90% specificity among blood donors and was used as the threshold for ELISA reactivity.

To further assess the specificity of antibody binding observed in ELISA assays using SE–containing peptides, an additional control immunoassay was performed using an unrelated peptide. Sera from patients with RA and control subjects were tested against a peptide derived from alpha-fetoprotein (AFP), corresponding to residues 365–384 (LAVSVILRVAKGYQELLEKC). This peptide does not share sequence homology with HLA-DRB1–derived peptides and does not contain the shared epitope motif. ELISA conditions, serum dilutions, and detection procedures were identical to those used for SE-derived peptides. The optical density values obtained were lower for RA patients than controls with mean ± SD values of 0.041 ± 0.023 and 0.059 ± 0.024, respectively, indicating the absence of reactivity against this unrelated peptide.

Laboratory analyses were performed blinded to clinical data and diagnostic group assignment.

### 2.5 Statistical analysis

Categorical variables were summarized as absolute frequencies and percentages, while continuous variables were presented as medians and ranges. Differences between patient groups in demographic and clinical characteristics were evaluated using the Mann-Whitney test for continuous variables and Fisher’s exact test for categorical variables. *Post-hoc* pairwise comparisons were adjusted for multiple comparisons with Holm correction.

As a sensitivity analysis, the discriminative performance of OD values was assessed using ROC analysis (RA vs controls; PsA vs controls), reporting AUC and classification metrics at the optimal threshold (closest-to-(0,1) criterion), with 95% confidence intervals obtained by bootstrap resampling (B = 1000).

Statistical significance was set at 5%. All analyses were performed using R 4.1.2 (R Core Team, 2021. R: A language and environment for statistical computing. R Foundation for Statistica).

## 3 Results

### 3.1 Patients and controls

A total of 150 RA patients treated at the outpatient clinics of PTUH were included in the study. The mean age at the time of participation was 63.8 years, with an average disease duration of 5.7 years. The mean age at diagnosis was 53.3 years, and the average time from symptom onset to diagnosis was approximately six months. Women represented 75.3% (n = 113) of the cohort. Overall, 65.4% (87) of RA patients were carriers of the SE, and 11.3% (n = 15) had a double SE load (**Supplementary Tables 1**, **S2**).

As an inflammatory joint disease control group, 62 patients with PsA were included. Among them, 37.9% (n = 11) were SE carriers, and 6.9% (n = 2) carried a double SE load (**Supplementary Table 2**). Additionally, 204 healthy controls were recruited from blood donors at the Banc de Sang i Teixits of Barcelona.

### 3.2 Association between autoantibodies against the shared epitope and the shared epitope

In RA patients, no association was identified between SE-AAb presence and SE carriage (**Table 1**). Sequence-specific SE analysis also showed no association between carriage and AAb presence (**Supplementary Table 3**, S4, and S5). However, SE carriage was significantly associated with RA serological markers, particularly ACPAs (p = 0.0015) (**Table 1**).

**Table 1:** HLA genotype for the shared epitope, presence of autoantibodies against the shared epitope peptide, and autoimmune markers in rheumatoid arthritis patients. The table examines the association between HLA genotype and ELISA positivity across sequences of the shared epitope peptide evaluated in this study (QKRAA, QRRAA, and RRRAA), considering different assay formats (cyclated or linear) and post-translational modifications (citrullination, carbamylation or no modification). The table also includes measurements for classical markers of rheumatic disease (Rheumatoid Factor, ACPA and ANA).

		HLA – SE carriers		
Autoantibodies	All N=133	Yes N=87 (65.4%)	No N=46 (34.6%)	P-value
Cyclated Citrullinated SE peptide Abs	56 (42.1%)	40 (46.0%)	16 (34.8%)	0.2686
Linear Citrullinated SE peptide Abs	42 (31.6%)	26 (29.9%)	16 (34.8%)	0.5634
Linear Carbamylated SE peptide Abs	20 (15.0%)	15 (17.2%)	5 (10.9%)	0.4463
Cyclated SE peptides Abs	34 (25.6%)	21 (24.1%)	13 (28.3%)	0.6773
Linear SE peptides Abs	36 (27.1%)	26 (29.9%)	10 (21.7%)	0.4124
Rheumatoid Factor	86 (64.7%)	61 (70.1%)	25 (54.3%)	0.0869
ACPA*	97 (74.0%)	71 (83.5%)	26 (56.5%)	**0.0015**
ANA*	42 (33.3%)	22 (27.5%)	20 (43.5%)	0.0791

*For the ACPA analysis, a total of 131 patients were included, of whom 13 were HLA-SE carriers and 118 were non-carriers. For ANA analysis, a total of 126 patients were included, of whom 80 were HLA-SE carriers and 46 were non-carriers.

Bold values correspond to results that are statistically significant (p < 0.05).

In PsA patients, the relationship between the presence of SE-AAb and being a carrier of SE was evaluated. Similar to the RA patients, no significant association was observed (**Supplementary Table 6**).

### 3.3 Antibodies against the shared epitope in RA, PsA, and controls

The performance of ELISA assays was evaluated in RA patients, comparing them with healthy blood donors and a cohort of PsA patients, used as a model of non-RA-related inflammatory arthritis. Compared to healthy donors, a significant presence of SE-AAb was observed in RA patients against all citrullinated sequences, both in cyclic and linear conformations, as well as against the carbamylated sequence. For non-citrullinated sequences, significance was only observed for the QRRAA polymorphism and the grouped sequences in its linear conformation (**Table 2**, **Supplementary Tables 7**, **S8**).

**Table 2:** Autoantibodies against shared epitope peptides by subject groups. The table displays the rate of positivity corresponding to the QKRAA, QRRAA, and RRRAA sequences, as well as across all of them (All), for linear and cyclated quantifications and considering different post-translational modifications: no-modification and citrullination or carbamylation.

Shared Epitope – AAbs		RA N=150 (36.1%)	PSA N=62 (14.9%)	Control N=204 (49.0%)	Overall P-value	Pairwise comparisons p-value		
						Control – RA	Control – PsA	RA – PSA
QKRAA	Cyclated Citrullinated SE peptides	37 (24.7%)	18 (29.0%)	20 (9.8%)	**<0.0001**	**0.0007**	**0.0012**	0.6056
	Linear Citrullinated SE peptides	35 (23.3%)	11 (17.7%)	21 (10.3%)	**0.0036**	**0.0033**	0.2447	0.4645
	Linear Carbamylated SE peptides	28 (18.7%)	7 (11.3%)	21 (10.3%)	0.0699	0.0873	0.8151	0.4516
	Cyclated SE peptides	16 (10.7%)	5 (8.1%)	21 (10.3%)	0.9033	>0.9999	>0.9999	>0.9999
	Linear SE peptides	16 (10.7%)	7 (11.3%)	21 (10.3%)	0.9757	>0.9999	>0.9999	>0.9999
QRRAA	Cyclated Citrullinated SE peptides	36 (24.0%)	20 (32.3%)	21 (10.3%)	**<0.0001**	**0.0014**	**0.0003**	0.2331
	Linear Citrullinated SE peptides	41 (27.3%)	16 (25.8%)	21 (10.3%)	**<0.0001**	**0.0002**	**0.0108**	0.8663
	Cyclated SE peptides	20 (13.3%)	7 (11.3%)	22 (10.8%)	0.7808	>0.9999	>0.9999	>0.9999
	Linear SE peptides	37 (24.7%)	14 (22.6%)	21 (10.3%)	**0.0007**	**0.0013**	**0.0353**	0.8602
RRRAA	Cyclated Citrullinated SE peptides	51 (34.0%)	15 (24.2%)	21 (10.3%)	**<0.0001**	**<0.0001**	**0.0193**	0.1930
	Linear Citrullinated SE peptides	36 (24.0%)	13 (21.0%)	21 (10.3%)	**0.0016**	**0.0021**	0.0961	0.7217
	Cyclated SE peptides	18 (12.0%)	5 (8.1%)	20 (9.8%)	0.6868	>0.9999	>0.9999	>0.9999
	Linear SE peptides	26 (17.3%)	2 (3.2%)	21 (10.3%)	**0.0083**	0.1171	0.1192	**0.0188**
All	Cyclated Citrullinated SE peptides	68 (45.3%)	22 (35.5%)	44 (21.6%)	**<0.0001**	**<0.0001**	0.0601	0.2224
	Linear Citrullinated SE peptides	51 (34.0%)	17 (27.4%)	28 (13.7%)	**<0.0001**	**<0.0001**	**0.0381**	0.4195
	Cyclated SE peptides	39 (26.0%)	11 (17.7%)	43 (21.1%)	0.3582	0.6541	0.7186	0.6541
	Linear SE peptides	45 (30.0%)	18 (29.0%)	28 (13.7%)	**0.0003**	**0.0008**	**0.0148**	>0.9999

Bold values correspond to results that are statistically significant (p < 0.05).

Interestingly, PsA patients exhibited a similar pattern to RA patients, with a significant presence of SE-AAb against citrullinated sequences in all three SE peptides, except for the linear conformation of the QKRAA polymorphism. For non-citrullinated sequences, significance was only observed for the QRRAA polymorphism and the grouped sequences in its linear conformation (**Table 2**).

When comparing the RA group with the PsA group, statistically significant differences were identified only for antibodies against the RRRAA polymorphism in its linear conformation (RA vs. PsA; 17.3% (n = 26) vs. 3.2% (n = 2), p = 0.0063) (**Table 2**).

### 3.4 Homology analysis between the shared epitope and the other HLA-DRB1 polymorphisms

Given the presence of SE-AAb in some RA and PsA patients who were not SE carriers, we analyzed the sequence similarity between their HLA-DRB1 alleles and the canonical SE motif. In our cohort, 34 RA and 13 PsA patients lacked SE alleles but tested positive for SE-AAb. Among these 47 patients, a total of 23 different HLA-DRB1 alleles were identified. Sequence homology between these alleles and the SE motif ranged from 60.0% to 93.3%. Notably, only one patient exhibited less than 80% homology between their alleles and the SE reference sequences. (**Supplementary Table 9**).

### 3.5 Association between autoantibodies against the shared epitope and clinical variables

The association between the presence of SE-AAb and the main clinical characteristics of RA was analyzed, but no significant relationship was found (**Table 3**).

**Table 3:** Patients’ characteristics by detection of autoantibodies against shared epitope peptides. The table shows the association between ELISA positivity across sequences of the shared epitope evaluated in this study (QKRAA, QRRAA and RRRAA) and patients’ clinical and demographic factors.

Patients’ characteristics		N	All N=150	SE-AAb Positive N=112 (74.7%)	SE-AAb Negative N=38 (25.3%)	P-value
Sex - Female		150	113 (75.3%)	87 (77.7%)	26 (68.4%)	0.2795
Age		150	63.8 (23.1, 91.7)	64.7 (23.1, 91.7)	61.0 (26.8, 88.8)	0.5169
Age at diagnosis		139	53.7 (19.2, 86.1)	50.2 (19.7, 86.0	54.4 (19.2, 86.1)	0.4594
RA duration time (years)		139	5.7 (0.0, 46.5)	5.7 (0.0, 46.5)	5.8 (0.1, 24.3)	0.7594
Time from symptom onset to diagnosis (years)		103	0.5 (0.0, 5.0)	0.5 (0.0, 5.0)	0.6 (0.0, 3.0)	0.6203
Smoking status	**Never**	143	77 (53.8%)	61 (56.5%)	16 (45.7%)	0.0969
	**Former**		46 (32.2%)	36 (33.3%)	10 (28.6%)	
	**Smoker**		20 (14.0%)	11 (10.2%)	9 (25.7%)	
Joint erosion disease		144	38 (26.4%)	30 (28.0%)	8 (21.6%)	0.5208
Rheumatoid factor		150	95 (63.3%)	67 (59.8%)	28 (73.7%)	0.1724
ACPA		148	108 (73.0%)	79 (71.8%)	29 (76.3%)	0.6751
ANA		142	47 (33.1%)	34 (32.1%)	13 (36.1%)	0.6850

## 4 Discussion

This study aimed to analyze the presence of SE-AAb in patients with RA. SE-AAb were detected in 26.0% (n = 39) to 45.3% (n = 68) of cases, depending on the conformation of the SE, with higher positivity observed for citrullinated forms. After confirming that the SE can act as a target for autoantibodies, we assessed whether SE-AAb positivity was associated with SE carriage. No significant association between these variables was observed.

One possible explanation for this finding relates to the methodology used to detect SE-AAb. The ELISA system may detect antibody binding through cross-reactivity, given the high degree of sequence homology among HLA-DRB1 polymorphisms within positions 66 to 78 (40, 41). These variants differ by only 1 to 6 amino acids within the 13–amino acid peptides used in the assay, which could facilitate antibody binding to SE-containing peptides. Consistent with this interpretation, sequence analysis of the HLA-DRB1 region in patients who were not SE carriers but tested positive for SE-AAb revealed a high degree of homology with the canonical SE sequence (41).

An alternative explanation is related to the broad autoreactive capacity observed in RA. Patients with RA can generate autoantibodies against multiple citrullinated proteins and peptides (9). Although SE carriage is associated with ACPA generation, ACPA positivity is also observed in patients who do not carry SE alleles, as citrullinated peptides can still be presented in the P4 pocket of HLA class II molecules (42).

Additional support for cross-reactive immune responses comes from studies using peptide libraries derived from SE-containing sequences. In those analyses, only the shared epitope showed significant homology with microbial proteins from *Proteus mirabilis*, *Escherichia coli*, *Brucella ovis*, *Salmonella dublin*, and other bacteria (43–47). These findings have led to the proposal that exposure to such microorganisms may induce SE-directed antibodies through mechanisms of molecular mimicry.

To assess the specificity of SE-AAb in relation to RA, a control group of PsA patients was included as a model of non–RA-related joint inflammation. In this group, SE-AAb were detected in 17.7% (n = 11) to 35.5% (n = 22) of cases. When compared with RA patients, no significant differences were observed, except for antibodies directed against the native linear RRRAA sequence.

Analysis of reactivity to native linear SE peptides revealed a progressive effect of arginine content. The presence of a single arginine did not increase reactivity beyond levels observed in healthy controls. In contrast, two arginine residues were associated with an approximately twofold increase in reactivity in both RA and PsA. Interestingly, the presence of a third arginine resulted in divergent patterns between the two diseases. Reactivity decreased mildly in RA but showed a more pronounced reduction in PsA. This may suggest that autoantibodies generated in PsA have a reduced capacity to bind peptides with a high density of positively charged residues. When arginines were citrullinated, peptide charge was altered and antibody binding was restored, reaching reactivity levels higher than those observed in healthy controls.

The relationship between SE-AAb positivity and SE carriage was also evaluated in PsA patients. Similar to the findings in RA, no significant association was observed. The detection of SE-AAb in PsA suggests that joint inflammation, regardless of its underlying cause, may contribute to the breakdown of immune tolerance (48). In this context, HLA-DRB1–derived peptides could act as targets of the autoimmune response.

Although immune dysregulation in PsA is primarily associated with the HLA class I system (49), which likely explains the lower prevalence of autoantibodies such as RF and ACPAs (5, 50, 51), increasing evidence supports a role for the adaptive immune system in this disease. Observations including lymphoid neogenesis and the presence of autoantibodies, particularly those directed against carbamylated peptides, support this concept (52–54). The detection of SE-AAb in our PsA cohort further reinforces this possibility.

Finally, we analyzed the association between SE-AAb positivity and clinical variables in RA using retrospectively collected data. No significant associations were identified. Due to the cross-sectional design of the study and the heterogeneity in disease duration, the influence of SE-AAb on clinical outcomes could not be assessed. An additional limitation is that the SE analyzed in this study corresponds only to the core 70–74 amino acid sequence. Other HLA-DRB1 positions, particularly residue 11, have been shown to independently and strongly influence RA susceptibility (55). Further prospective studies will therefore be required to determine whether SE-AAb have clinical relevance in RA.

In conclusion, SE-AAb are present in RA, particularly against citrullinated shared epitope peptides, but are not disease-specific and occur at similar frequencies in PsA. Their presence is independent of shared epitope genetic carriage, suggesting that inflammatory context rather than genetic risk alone may drive these autoantibody responses.

## Data Availability

The original contributions presented in the study are included in the article/supplementary material. Further inquiries can be directed to the corresponding authors.
