# Supplementary material for: Presence of autoantibodies targeting the shared epitope in rheumatoid arthritis and psoriatic arthritis
Source: Front Immunol. 2026 Mar 3;17:1744370. doi: 10.3389/fimmu.2026.1744370 (PMC13019701; doi:10.3389/fimmu.2026.1744370)
Supplement: Supplementary file 1 [file Supplementaryfile1.docx]

**Supplementary Table S1.** **Descriptive statistics of patients with Rheumatoid Arthritis (RA) included in the study.** The table displays the main demographic variables, clinical factors, HLA genotype for the shared epitope in three different sequences (QKRAA, QRRAA, and RRRAA), and the number of allele copies across the three sequences (dose). Continuous parameters are described with their medians and ranges (minimum and maximum values), while absolute frequencies and percentages are provided for categorical variables, considering patients with available data (N available). **csDMARD**: Classical synthetic DMARD

|  | | **N Available** | **All N=150** | **Female N=113 (75.3%)** | **Male N=37 (24.7%)** | **P-value** |
| --- | --- | --- | --- | --- | --- | --- |
| **Age** | | 150 | 63.8 (23.1, 91.7) | 62.5 (23.1, 91.7) | 65.6 (36.2, 88.8) | 0.5287 |
| **Age at diagnosis** | | 139 | 53.7 (19.2, 86.1) | 53.6  (19.2, 86.1) | 57.3  (19.7, 86.0) | 0.2613 |
| **RA duration time  (years)** | | 139 | 5.7 (0.0, 46.5) | 6.0 (0.0, 46.5) | 5.1 (0.1, 24.3) | 0.4314 |
| **Time from symptom onset to diagnosis  (years)** | | 103 | 0.5  (0.0, 5.0) | 0.5  (0.0, 5.0) | 0.4  (0.0, 3.3) | 0.7248 |
| **Smoking status** | **Never** | 143 | 77 (53.8%) | 68 (62.4%) | 9 (26.5%) | **0.0006** |
|  | **Former** |  | 46 (32.2%) | 27 (24.8%) | 19 (55.9%) |  |
|  | **Smoker** |  | 20 (14.0%) | 14 (12.8%) | 6 (17.6%) |  |
| **Treatment line** | **Glucocorticoids only / csDMARDs** | 149 | 108 (72.5%) | 83 (73.5%) | 25 (69.4%) | 0.8498 |
|  | **First-line advanced DMARDs** |  | 30 (20.1%) | 22 (19.5%) | 8 (22.2%) |  |
|  | **Second-line or further, of advanced DMARDs** |  | 11 (7.4%) | 8 (7.1%) | 3 (8.3%) |  |
| **Erosive joint disease** | | 144 | 38 (26.4%) | 30 (27.5%) | 8 (22.9%) | 0.6640 |
| **Rheumatoid factor** | | 150 | 95 (63.3%) | 72 (63.7%) | 23 (62.2%) | >0.9999 |
| **ACPA** | | 148 | 108 (73.0%) | 81 (73.0%) | 27 (73.0%) | >0.9999 |
| **Antinuclear Antibody (ANA)** | | 142 | 47 (33.1%) | 35 (32.7%) | 12 (34.3%) | >0.9999 |
| **QKRAA genotype** | | 133 | 13 (9.8%) | 9 (9.2%) | 4 (11.4%) | 0.7433 |
| **QRRAA genotype** | | 133 | 73 (54.9%) | 48 (49.0%) | 25 (71.4%) | **0.0292** |
| **RRRAA genotype** | | 133 | 11 (8.3%) | 8 (8.2%) | 3 (8.6%) | >0.9999 |
| **Shared epitope dose** | **0 copies** | 133 | 46 (34.6%) | 39 (39.8%) | 7 (20.0%) | 0.0726 |
|  | **1 copy** |  | 72 (54.1%) | 50 (51.0%) | 22 (62.9%) |  |
|  | **2 copies** |  | 15 (11.3%) | 9 (9.2%) | 6 (17.1%) |  |

**Supplementary Table S2. Genotype for the shared epitope in patients with rheumatic conditions.** The table shows the frequencies and percentages of the QKRAA, QRRAA, and RRRAA sequences in all subjects, as well as in patients with psoriatic arthritis (PSA) and rheumatoid arthritis (RA).

| **Genotype** | **All n=162** | **PSA n=29** | **RA n=133** | **P-value** |
| --- | --- | --- | --- | --- |
| **QKRAA** | 14 (8.6%) | 1 (3.4%) | 13 (9.8%) | 0.4679 |
| **QRRAA** | 83 (51.2%) | 10 (34.5%) | 73 (54.9%) | 0.0642 |
| **RRRAA** | 11 (6.8%) | 0 (0.0%) | 11 (8.3%) | 0.2161 |
| **SE carrier** | 98  (60.5%) | 11  (37.9%) | 87  (65.4%) | 0.0109 |
| **SE 0 copies** | 64  (39.5%) | 18  (62.1%) | 46  (34.6%) | 0.0253 |
| **SE 1 copy** | 81  (50.0%) | 9  (31.0%) | 72  (54.1%) |  |
| **SE 2 copies** | 17  (10.5%) | 2  (6.9%) | 15  (11.3%) |  |

**Supplementary Table S3. HLA Genotype for the shared epitope for sequence QKRAA, presence of autoantibodies against QKRAA sequence, and autoimmune markers.** The table examines the association between HLA genotype and ELISA positivity for sequence QKRAA, considering different assay formats (cyclated or linear) and post-translational modifications (citrullination, carbamylation or no modification). The table also includes measurements for classical markers of rheumatic disease (rheumatoid factor, ACPA and ANA).

* For the ACPA analysis, a total of 131 patients were included, of whom 13 were HLA-SE carriers and 118 were non-carrieres. For ANA analysis, a total of 126 patients were included, of whom 80 were HLA-SE carriers and 46 were non-carriers.

|  |  | **HLA – QKRAA** | |  |
| --- | --- | --- | --- | --- |
|  | **All N=133** | **Yes N=13 (9.8%)** | **No N=120 (90.2%)** | **P-value** |
| **Cyclated Citrullinated SE peptides** | 28 (21.1%) | 3 (23.1%) | 25 (20.8%) | >0.9999 |
| **Linear Carbamylated SE peptides** | 20 (15.0%) | 2 (15.4%) | 18 (15.0%) | >0.9999 |
| **Linear Citrullinated SE peptides** | 28 (21.1%) | 1 (7.7%) | 27 (22.5%) | 0.2992 |
| **Cyclated SE peptides** | 14 (10.5%) | 3 (23.1%) | 11 (9.2%) | 0.1405 |
| **Linear SE peptides** | 12 (9.0%) | 1 (7.7%) | 11 (9.2%) | >0.9999 |
| **Rheumatoid Factor** | 86 (64.7%) | 11 (84.6%) | 75 (62.5%) | 0.1371 |
| **ACPA*** | 97 (74.0%) | 12 (92.3%) | 85 (72.0%) | 0.1820 |
| **ANA*** | 42 (33.3%) | 3 (23.1%) | 39 (34.5%) | 0.5415 |

**Supplementary Table S4. HLA Genotype for the shared epitope for sequence QRRAA, presence of autoantibodies against QRRAA sequence, and autoimmune markers.** The table examines the association between HLA genotype and ELISA positivity for sequence QRRAA, considering different assay formats (cyclated or linear) and post-translational modifications (citrullination or no modification). The table also includes measurements for classical markers of rheumatic disease (rheumatoid factor, ACPA and ANA).

* For the ACPA analysis, a total of 131 patients were included, of whom 13 were HLA-SE carriers and 118 were non-carrieres. For ANA analysis, a total of 126 patients were included, of whom 80 were HLA-SE carriers and 46 were non-carriers.

|  |  | **HLA – QRRAA** | |  |
| --- | --- | --- | --- | --- |
|  | **All N=133** | **Yes N=73 (54.9%)** | **No N=60 (45.1%)** | **P-value** |
| **Cyclated Citrullinated SE peptides** | 25 (18.8%) | 14 (19.2%) | 11 (18.3%) | >0.9999 |
| **Linear Citrullinated SE peptides** | 32 (24.1%) | 18 (24.7%) | 14 (23.3%) | >0.9999 |
| **Cyclated SE peptides** | 19 (14.3%) | 8 (11.0%) | 11 (18.3%) | 0.3195 |
| **Linear SE peptides** | 29 (21.8%) | 17 (23.3%) | 12 (20.0%) | 0.6788 |
| **Rheumatoid Factor** | 86 (64.7%) | 52 (71.2%) | 34 (56.7%) | 0.1013 |
| **ACPA*** | 97 (74.0%) | 60 (84.5%) | 37 (61.7%) | **0.0047** |
| **ANA*** | 42 (33.3%) | 21 (31.8%) | 21 (35.0%) | 0.7102 |

**Supplementary Table S5. HLA Genotype for the shared epitope for sequence RRRAA, presence of autoantibodies against RRRAA sequence, and autoimmune markers.** The table examines the association between HLA genotype and ELISA positivity for sequence RRRAA, considering different assay formats (cyclated or linear) and post-translational modifications (citrullination or no modification). The table also includes measurements for classical markers of rheumatic disease (rheumatoid factor, ACPA and ANA).

* For the ACPA analysis, a total of 131 patients were included, of whom 13 were HLA-SE carriers and 118 were non-carrieres. For ANA analysis, a total of 126 patients were included, of whom 80 were HLA-SE carriers and 46 were non-carriers.

|  |  | **HLA – RRRAA** | |  |
| --- | --- | --- | --- | --- |
|  | **All N=133** | **Yes N=11 (8.3%)** | **No**  **N=122 (91.7%)** | **P-value** |
| **Cyclated Citrullinated SE peptides** | 41 (30.8%) | 6 (54.5%) | 35 (28.7%) | 0.0931 |
| **Linear Citrullinated SE peptides** | 33 (24.8%) | 2 (18.2%) | 31 (25.4%) | 0.7309 |
| **Cyclated SE peptides** | 15 (11.3%) | 1 (9.1%) | 14 (11.5%) | >0.9999 |
| **Linear SE peptides** | 23 (17.3%) | 3 (27.3%) | 20 (16.4%) | 0.4028 |
| **Rheumatoid Factor** | 86 (64.7%) | 7 (63.6%) | 79 (64.8%) | >0.9999 |
| **ACPA*** | 97 (74.0%) | 9 (81.8%) | 88 (73.3%) | 0.7274 |
| **ANA*** | 42 (33.3%) | 0 (0.0%) | 42 (36.2%) | **0.0300** |

**Supplementary Table S6. HLA Genotype for the shared epitope and protein presence in Psoriatic Arthritis patients.** The table examines the association between HLA genotype and ELISA positivity across sequences of the shared epitope evaluated in this study (QKRAA, QRRAA, and RRRAA), considering different assay formats (cyclated or linear) and post-translational modifications (citrullination or no modification). The table also includes measurements for classical markers of rheumatic disease activity.

|  |  | **HLA – SE carriers** | |  |
| --- | --- | --- | --- | --- |
|  | **All N=29** | **No N=18 (62.1%)** | **Yes N=11 (37.9%)** | **P-value** |
| **Cyclated Citrullinated SE peptides** | 14 (48.3%) | 9 (50.0%) | 5 (45.5%) | >0.9999 |
| **Linear Citrullinated SE peptides** | 11 (37.9%) | 6 (33.3%) | 5 (45.5%) | 0.6965 |
| **Linear Carbamylated SE peptides** | 2 (6.9%) | 0 (0.0%) | 2 (18.2%) | 0.1355 |
| **Cyclated SE peptides** | 6 (20.7%) | 5 (27.8%) | 1 (9.1%) | 0.3623 |
| **Linear SE peptides** | 13 (44.8%) | 8 (44.4%) | 5 (45.5%) | >0.9999 |

**Supplementary Table S7.**  **Autoantibodies against shared epitope peptides by subject groups.** The table displays optical density (OD) value distributions corresponding to the QKRAA, QRRAA and RRRAA sequences for linear and cyclated quantifications and considering different post-translational modifications: no-modification and citrullination or carbamylation. OD distributions are summarized as median (minimum, maximum) for each sequence and group. Overall differences among groups were assessed using Kruskal-Wallis test. Pairwise comparisons were performed using Mann-Whitney test, with Holm correction to adjust for multiple comparisons.

| **Shared Epitope – AAbs** | | **RA N=150 (36.1%)** | **PSA N=62 (14.9%)** | **Control N=204**  **(49.0%)** | **Overall P-value** | **Pairwise comparisons p-value** | | |
| --- | --- | --- | --- | --- | --- | --- | --- | --- |
|  |  |  |  |  |  | **Control – RA** | **Control – PsA** | **RA – PSA** |
| **QKRAA** | **Cyclated Citrullinated SE peptides** | 0.13 (0.00,0.80) | 0.15 (0.05,0.66) | 0.14 (0.00,0.33) | 0.0809 | 0.6824 | 0.0702 | 0.1549 |
|  | **Linear Citrullinated SE peptides** | 0.12 (0.00,1.84) | 0.09 (0.01,1.33) | 0.09 (0.01,0.70) | **0.0007** | **0.0009** | 0.6462 | **0.0234** |
|  | **Linear Carbamylated SE peptides** | 0.12 (0.02,0.89) | 0.08 (0.02,1.16) | 0.10 (0.01,1.48) | **0.0062** | **0.0198** | 0.3109 | **0.0230** |
|  | **Cyclated SE peptides** | 0.10 (0.00,1.30) | 0.10 (0.00,0.60) | 0.09 (0.00,1.80) | 0.8799 | >0.9999 | >0.9999 | >0.9999 |
|  | **Linear SE peptides** | 0.08 (0.00,1.83) | 0.06 (0.00,0.66) | 0.06 (0.00,2.50) | 0.1575 | 0.2025 | 0.9256 | 0.3968 |
| **QRRAA** | **Cyclated Citrullinated SE peptides** | 0.14 (0.00,1.81) | 0.16 (0.04,0.77) | 0.13 (0.00,0.65) | **0.0019** | 0.1507 | **0.0009** | 0.0566 |
|  | **Linear Citrullinated SE peptides** | 0.13 (0.00,1.79) | 0.12 (0.01,1.06) | 0.10 (0.00,0.74) | **0.0261** | **0.0191** | 0.5203 | 0.4906 |
|  | **Cyclated SE peptides** | 0.14 (0.00,0.70) | 0.13 (0.04,0.64) | 0.12 (0.02,0.30) | 0.4337 | 0.8635 | 0.8635 | 0.6308 |
|  | **Linear SE peptides** | 0.12 (0.00,6.22) | 0.10 (0.00,0.79) | 0.10 (0.00,5.93) | 0.1412 | 0.1337 | 0.7579 | 0.7197 |
| **RRRAA** | **Cyclated Citrullinated SE peptides** | 0.16 (0.00,0.72) | 0.14 (0.00,0.94) | 0.14 (0.00,0.37) | **0.0050** | **0.0019** | 0.6344 | 0.5065 |
|  | **Linear Citrullinated SE peptides** | 0.14 (0.02,3.96) | 0.12 (0.01,0.81) | 0.11 (0.01,0.71) | **0.0021** | **0.0046** | 0.4021 | **0.0199** |
|  | **Cyclated SE peptides** | 0.14 (0.00,0.52) | 0.11 (0.00,0.29) | 0.13 (0.00,1.68) | 0.6720 | >0.9999 | >0.9999 | 0.9463 |
|  | **Linear SE peptides** | 0.07 (0.00,3.16) | 0.06 (0.00,1.28) | 0.05 (0.00,2.04) | 0.0577 | 0.0624 | 0.4123 | 0.6112 |

**
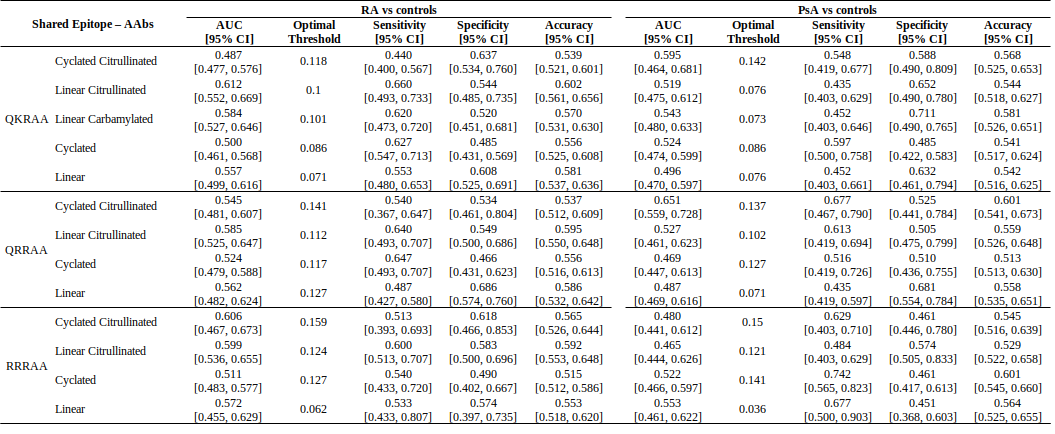
Supplementary Table S8**. Discriminative performance of autoantibodies against shared epitope peptides in RA and PsA versus controls.
Receiver operating characteristic (ROC) analysis was performed on optical density (OD) values for each autoantibody quantification to assess discrimination between rheumatoid arthritis (RA) vs controls and psoriatic arthritis (PsA) vs controls. For each comparison, the area under the curve (AUC), sensitivity, specificity, and balanced accuracy are reported at the optimal threshold, defined as the point closest to the upper-left corner of the ROC space (closest-to-(0,1) criterion). Estimates are shown together with 95% confidence intervals, obtained by stratified bootstrap resampling (B = 1000). Balanced accuracy was calculated as (sensitivity + specificity)/2.

**Supplementary Table S9.** **Structural homology between HLA-DRB1 polymorphisms and SE-related sequences.** The structure of HLA-DRB1 polymorphisms was analyzed in patients who tested positive for SE-AAb despite not carrying the shared epitope. The table displays the degree of structural homology between these HLA-DRB1 variants and the canonical SE-associated polymorphisms.

| Alleles | Aminoacid diferences with QKRAA peptide | Aminoacid diferences with QRRAA peptide | Aminoacid diferences with RRRAA peptide | % homology with QKRAA peptide | % homology with RKRAA peptide | % homology with RRRAA peptide |
| --- | --- | --- | --- | --- | --- | --- |
| 01:03 | 3 | 3 | 3 | 80,0 | 80,0 | 80,0 |
| 03:01 | 3 | 4 | 5 | 80,0 | 73,3 | 66,7 |
| 04:02 | 3 | 3 | 3 | 80,0 | 80,0 | 80,0 |
| 04:03 | 2 | 1 | 2 | 86,7 | 93,3 | 86,7 |
| 07:01 | 6 | 5 | 6 | 60,0 | 66,7 | 60,0 |
| 08:01 | 4 | 3 | 3 | 73,3 | 80,0 | 80,0 |
| 08:06 | 4 | 3 | 3 | 73,3 | 80,0 | 80,0 |
| 09:01 | 5 | 4 | 3 | 66,7 | 73,3 | 80,0 |
| 11:01 | 3 | 2 | 2 | 80,0 | 86,7 | 86,7 |
| 11:02 | 3 | 3 | 3 | 80,0 | 80,0 | 80,0 |
| 11:03 | 3 | 3 | 3 | 80,0 | 80,0 | 80,0 |
| 11:04 | 3 | 2 | 2 | 80,0 | 86,7 | 86,7 |
| 13:01 | 3 | 3 | 3 | 80,0 | 80,0 | 80,0 |
| 13:02 | 3 | 3 | 3 | 80,0 | 80,0 | 80,0 |
| 13:03 | 2 | 3 | 3 | 86,7 | 80,0 | 80,0 |
| 13:04 | 3 | 3 | 3 | 80,0 | 80,0 | 80,0 |
| 13:05 | 3 | 2 | 2 | 80,0 | 86,7 | 86,7 |
| 14:01 | 3 | 2 | 1 | 80,0 | 86,7 | 93,3 |
| 15:01 | 2 | 2 | 3 | 86,7 | 86,7 | 80,0 |
| 15:02 | 2 | 2 | 3 | 86,7 | 86,7 | 80,0 |
| 15:14 | 2 | 2 | 3 | 86,7 | 86,7 | 80,0 |
| 16:01 | 3 | 2 | 2 | 80,0 | 86,7 | 86,7 |
| 16:02 | 2 | 1 | 1 | 86,7 | 93,3 | 93,3 |
